# The Levels of Mineral Elements and Toxic Metals in the *Longissimus lumborum* Muscle, Hair and Selected Organs of Red Deer (*Cervus elaphus* L.) in Poland

**DOI:** 10.3390/ani11051231

**Published:** 2021-04-24

**Authors:** Dorota Cygan-Szczegielniak

**Affiliations:** Department of Animal Physiology and Physiotherapy, Faculty of Animal Breeding and Biology, UTP University of Science and Technology, Mazowiecka 28, 85-004 Bydgoszcz, Poland; cygan@utp.edu.pl

**Keywords:** red deer, tissues, organs, mineral elements, toxic metals

## Abstract

**Simple Summary:**

The relationships between nutritive elements (Fe, Mn, Zn, Cu, Na, K, Ca and Mg) and toxic metals (Pb and Cd) can inform the assessment of health status of red deer, which are exposed to pollution in their natural environment. These elements in the *longissimus lumborum* muscle, hair and selected organs of red deer (*Cervus elaphus* L.) were analysed. The study revealed many significant correlations between the levels of elements and their high variability in individual tissues and organs. The accumulation of a specific metal in a tissue or organ can reduce its concentration in another matrix or promote increased content in another tissue or organ.

**Abstract:**

The aim of the study was to analyse correlations and variability between the levels of mineral elements (Fe, Mn, Zn, Cu, Na, K, Ca and Mg) and toxic metals (Pb and Cd) in the *longissimus lumborum* muscle, hair and selected organs of red deer (*Cervus elaphus* L.). The levels of mineral elements were measured using atomic absorption spectroscopy (F-AAS). The levels of Pb and Cd were analysed by means of electrothermal atomic absorption spectroscopy (ET-AAS). Of all analysed microelements, the highest concentration (in g·kg^−1^ of dry weight) was found for Fe in the liver. Considering all macroelements, the highest level was found for K in the *longissimus lumborum* muscle. Particularly remarkable was the high level of Pb in the muscle and hair of red deer. The level of Cd in the hair was four to five times lower than in other samples. The study revealed many significant correlations between the levels of certain elements in individual tissues and organs. There were significant correlations between Cu-Fe (r_xy_ = 0.650; *p* ≤ 0.01), Pb-Cu (r_xy_ = −0.863; *p* ≤ 0.01) and Cd-Ca (r_xy_ = −0.743; *p* ≤ 0.01) in testicles. For kidneys, a significant negative correlation was found for K and Na (r_xy_ = −0.779; *p* ≤ 0.01) and for Ca and Cd (r_xy_ = −0.695; *p* ≤ 0.01), and a positive correlation between Cu and Mn (r_xy_ = 0.693; *p* ≤ 0.01). In the liver, significant negative correlations were found between Ca-K (r_xy_ = −0.654; *p* ≤ 0.05), Cd-Fe (r_xy_ = −0.651; *p* ≤ 0.05) and Pb-Cu (r_xy_ = −0.627; *p* ≤ 0.05). In the muscle, only single significant correlations were found between Cd and Mn (r_xy_ = 0.648; *p* ≤ 0.05). The highest number of significant correlations were recorded for the hair, among others between Na-K (r_xy_ = 0.760) and Ca-Zn (r_xy_ = 0.709) at *p* ≤ 0.01. The study revealed high variability in the levels of mineral elements and selected toxic metals in tissues, organs and hair of red deer. Additionally, this research confirmed that the accumulation of a specific metal in a tissue or organ can reduce its concentration in another matrix or promote its increased content in another tissue or organ.

## 1. Introduction

Macroelements and microelements are essential nutrients for the proper functioning of all living organisms. Interactions between these elements, which may be synergistic or antagonistic, are also very important. Deficiency, excess or disturbed homeostasis of these elements in the animal body, triggered by biological, genetic or environmental factors, may be among the causes of various physiological dysfunctions. Minerals such as Cu, Co, Mn and Zn are particularly important for ruminants, and their deficiencies can lead to serious problems with reproduction, impair growth and also cause osteochondrosis [1]. Major factors which may lead to the onset of certain disorders in the mineral status include environmental pollution manifested by the contamination of soil, plants, water or air, which is often associated with biogeochemical conditions [2]. Appropriate concentrations of micro- and macroelements and their ratios in tissues, body fluids and in the hair of animals determine the normal growth and health of animals. Considering game species, the main problem is the accumulation of toxic metals, including Pb and Cd, in animal tissues. The monitoring of toxic metals in specific organs can provide valuable information about pollution of animal habitats, and about their exposure to these pollutants [2,3]. Both xenobiotics, Pb and Cd, enter living organisms via two major routes: enteral and respiratory [3], and due to their high mobility in the natural environment and strong tendency to accumulate, they undergo biomagnification in the food chain [4]. Game species spend their whole life in their natural habitats, and therefore, their tissues can be used as excellent indicators for the measurement of local pollution levels [5,6,7,8,9]. The transfer of elements from soil to plants and animals also depends on soil conditions, i.e., redox potential, pH or the content, of organic matter and plant physiology [10,11,12]. The higher the pH and the greater the content of organic matter in the soil, the more the elements are associated with its surface layer (they are not leached into deeper layers), which in turn promotes easier physiological uptake and accumulation of the elements [10]. Plants are particularly exposed to the serious threat posed by various pollutants, such as heavy metals, which enter them from the soil together with water and air. For plants, Pb is one of the most toxic metals [12]. The concentration of elements in plants differs depending on the ecotype, forest vs. field [7]. Some plants tend to accumulate more pollutants [10] of which the consumption by an animal is a source of direct exposure to these compounds and their increased accumulation in animal tissues [7]. Toxic elements can accumulate in hard tissues, such as bones, teeth [7,8] and antlers [9], in hair [3,5,13,14] and in the parenchymal organs, such as the liver [4,15,16], muscles [6,17] and kidneys [4,18]. Red deer living in a given area have different access to essential trace elements and toxic elements, which is reflected in their different concentration in animal tissues [11]. Zn is concentrated mainly in the liver and distributed throughout the body. In addition to the liver, the main storage sites for Zn are bones, pancreas, kidneys and muscles [10]. Zn and Cu are elements essential for the health and growth of animals; their toxic effects can result from both deficiency and excess [18,19]. Zn is a component of regulatory proteins and DNA binding proteins, while Cu is a component of many enzymes [20]. When Cu from the gastrointestinal tract reaches the bloodstream, it binds to albumin and histidine, after which it is transported to the liver via the portal circulation. Next, Cu forms complexes with cytoplasmic proteins in the hepatic cells. The liver and kidneys are the organs in which the concentration of cadmium after absorption reaches the highest values. On the other hand, Pb is distributed throughout the body and, regardless of the way of absorption, deposited mainly in the bones [10]. In the body of mammals, Pb also accumulates in the liver and kidneys [15].

The aim of the study was to analyse correlations and variability between the levels of mineral elements (Fe, Mn, Zn, Cu, Na, K, Ca and Mg) and toxic metals (Pb and Cd) in the *longissimus lumborum* muscle, hair and selected organs of red deer (*Cervus elaphus* L.).

## 2. Materials and Methods

### 2.1. Study Material

The study material comprised testicles, liver, kidneys, the *longissimus lumborum* muscle and hair collected from red deer (*Cervus elaphus* L.) in the Kuyavian-Pomeranian province. The Kuyavian-Pomeranian province, located in the central part of Poland, is characterised by a moderate degree of industrialisation. The major potential emitters of pollutions to the environment in this area are the chemical industry, electrical and machinery industries, food processing plants, print shops and the cellulose industry. Levels of cadmium, nickel and arsenic (for which target values exist), measured in particulate matter (PM10), and levels of Pb reported from all monitoring stations were within the acceptable limits for all these metals [21]. Samples were collected from 20 stags. Deer were aged approximately 4–6 years. The age of animals was estimated based on the degree of tooth wear [22]. The animals were shot by hunters in hunting districts within the Kuyavian-Pomeranian region in compliance with the Regulation of the Ministry of Environment of 16 March 2005 (Journal of Laws, No. 48, item 459). Stags were shot between 21 August and the end of February, in compliance with the above-mentioned regulation. Due to the fact that the animals were obtained from their natural habitat, where the quantitative and qualitative composition of the vegetation eaten by them was impossible to control, the chemical composition of red deer diet was not analysed.

### 2.2. Sample Preparation

Hair samples (*n =* 20) were collected directly behind the arcus costalis, using stainless-steel laboratory scissors. This sampling technique eliminated the risk of chemical contamination. Hair samples were dissected close to the skin from a 10 × 10 cm area, and kept in a sealed polyethylene bag in a dry and shaded place until further analysis. To remove dirt and grease, samples were washed with acetone and placed for 15 min in an ultrasonic cleaner. Samples were stored for 12 h. Acetone was removed by decantation, after which the hair was rinsed twice with distilled water and dried in an oven at a temperature not exceeding 50 °C. The appropriate procedure for preparing the hair for analysis ensured that it was free from potential superficial chemical contamination.

Samples of the *longissimus lumborum* muscle (*n =* 20) from the segment along the first three lumbar vertebrae, testicles (*n =* 20), liver (*n =* 20) and kidneys (*n =* 20) were also collected for analysis. These samples were lyophilised in a Lyovac GT2 freeze dryer (Finn-Aqua). All samples (including hair) were then wet-digested using an EthosPlus microwave digestion system (Milestone), according to Polish Standard PN-EN 13805: 2014 [23]. For this purpose, 0.20 g aliquots of all analysed samples were prepared and then treated with 6.25 cm^3^ of a mixture of 65% HNO_3_ and 30% H_2_O_2_ in a 4:1 volume ratio (v:v). Digestion time was 20 min. Within the first 10 min, the temperature was gradually increased to 190 °C, and then maintained at 190 °C ± 5 °C. After completed digestion samples were transferred to 25 mL volumetric flasks and the volume was adjusted with distilled water. The concentration of mineral elements in the analysed samples was expressed in g·kg^−1^ and mg·kg^−1^ of dry weight (d.w.). The concentration of iron (Fe), manganese (Mn), zinc (Zn), copper (Cu), sodium (Na), potassium (K), calcium (Ca) and magnesium (Mg) was measured by means of atomic absorption spectroscopy (F-AAS), and the concentration of lead (Pd) and cadmium (Cd) was determined using electrothermal atomic absorption spectroscopy (ET-AAS). Analyses were conducted at a certified laboratory in compliance with procedures proposed by Chatt and Katz [24] and the Polish Standard PN-EN-14084:2004 [25].

### 2.3. Statistical Analyses

Some statistics did not meet the requirements for normal distribution (which was verified using the Shapiro–Wilk test) or requirements for the homogeneity of variance which are necessary to run parametric tests. Therefore, the significance of differences between experimental groups was analysed with non-parametric tests. The significance of differences between many independent samples (research matrices) was analysed with non-parametric tests (non-parametric ANOVA), i.e., the ANOVA Kruskal–Wallis and the median test. Additionally, the post-hoc for ANOVA Kruskal–Wallis test was performed, i.e., the multiple comparison of mean ranks for all samples. Correlations between selected parameters were analysed using Spearman’s rank correlation coefficients. The coefficient of variation (CV) was also calculated, demonstrating the differentiation of the feature in a given matrix. The obtained data were processed using Statistica 13.1 software.

## 3. Results

Table 1, Table 2 and Table 3 present the concentrations of microelements, macroelements and toxic metals in individual tissues, organs and hair of red deer. Of all the analysed microelements, the highest concentration (in g·kg^−1^ of d.w.) was found for Fe. The highest concentration of Fe was measured in the liver. Many significant differences between the concentrations of individual microelements were found, as indicated in detailed data presented in Table 1. The level of Mn ranged from 6.57 mg·kg^−1^ in testicles to 19.32 mg·kg^−1^ in hair. Levels of Zn differed largely between organs, from 43.2 mg·kg^−1^ in the liver to 144 mg·kg^−1^ in hair. Levels of Cu ranged from 16.7 in kidneys to 43.4 mg·kg^−1^ in the longissimus lumborum muscle. Concentrations of the analysed microelements, regardless of tissue or organ, were in the order from highest to lowest as follows: Fe > Zn > Cu > Mn. The highest variability among all microelements was observed for Fe in the hair, and the lowest for Cu in the liver (Table 1). 

Considering all macroelements, the highest level was found for K in the *longissimus lumborum* muscle. There were many significant differences between tissues and organs in the levels of individual macroelements, as indicated in detailed data presented in Table 2. The level of sodium was lowest in the liver (0.94 g·kg^−1^) and highest in testicles (3.6 g·kg^−1^). K levels in organs and tissues differed considerably, from 1.15 in the hair to 14.06 g·kg^−1^ in the *longissimus lumborum* muscle. Levels of Ca ranged from 0.11 in kidneys to 1.8 in the hair, and levels of Mg were from 0.15 in the liver to 1.43 in the *longissimus lumborum* muscle. Concentrations of the analysed macroelements, regardless of tissue or organ, were in the order from highest to lowest as follows: K > Na > Mg > Ca. The highest variability among all macroelements was observed for Ca in the kidneys, and the lowest for K in the liver (Table 2). 

Table 3 presents the content of toxic metals in individual samples. Particularly remarkable was the high level of Pb in the *longissimus lumborum* muscle and hair of red deer. The levels of Pb ranged from 2.59 in the liver to 7.57 mg·kg^−1^ in the hair. Moreover, the content of Cd in the hair was 4–5 times lower than in other organs and ranged from 0.13 in the hair to 0.56 mg·kg^−1^ in the liver. The highest variability among toxic metals was observed for Pb in the muscle, and the lowest for Cd in the testicles (Table 3).

The coefficients of correlation between the selected elements and their variability in individual samples were also calculated. Due to the large number of statistically insignificant correlations, Table 4 presents only significant correlations. Interestingly, significant correlations (positive or negative) between the *longissimus lumborum* muscle/organ, organ/hair or organ/organ system were found only for certain elements. In the case of kidneys, a statistically significant correlation was found mainly with hair for Fe, Mg, Mn and Cu, and the *longissimus lumborum* muscle for Ca, while in the case of liver only with hair for Pb and Cd and with kidneys for Na. For the remaining elements, no statistically significant correlations were found between the different matrices. The following data illustrate the correlations between the concentrations of metal in two different biological matrices (samples).

The highest number of significant interactions were recorded for the hair, and statistics for this biological matrix are presented in Table 5. The number of correlations established for other tissues or organs was much lower, and statistics are presented in Table 6, Table 7, Table 8 and Table 9. In the hair, highly statistically significant correlations, among others, were found between Na and K (r_xy_ = 0.760; *p* ≤ 0.01) and Ca and Zn (r_xy_ = 0.709; *p* ≤ 0.01), as well as significant correlations at *p* ≤ 0.05 between Fe and Cu and Mn and Cu of r_xy_ = 0.687 and r_xy_ = 0.693 and one negative statistically significant correlation between Zn and Cu (r_xy_= −0.682; *p* ≤ 0.05). There was a significant correlation between Cu-Fe (r_xy_ = 0.650; *p* ≤ 0.01), Pb-Cu (r_xy_= −0.863; *p* ≤ 0.01) and Cd-Ca (r_xy_= −0.743; *p* ≤ 0.01) in testicles. For kidneys, a significant negative correlation was found for K and Na (r_xy_ = −0.779; *p* ≤ 0.01) and Ca and Cd, (r_xy_ = −0.695; *p* ≤ 0.01), and a positive correlation between Cu and Mn (r_xy_ = 0.693; *p* ≤ 0.01). In the liver, significant negative correlations were found between Ca-K (r_xy_ = −0.654; *p* ≤ 0.05), Cd-Fe (r_xy_ = −0.651; *p* ≤ 0.05) and between Pb-Cu (r_xy_ = −0.627; *p* ≤ 0.05). In the muscle, only single significant correlations were found between Cd and Mn (r_xy_ = 0.648; *p* ≤ 0.05).

## 4. Discussion

The study revealed high variability in the levels of mineral elements (Table 1 and Table 2) and selected toxic metals in tissues, organs and hair of red deer (Table 3). This is probably strongly influenced by differences in the composition of these anatomical structures and their physiological functions, type of fibre, and thus, different degrees of biotransformation of these elements, as well as environmental factors [26]. Significant correlations between the concentrations of some elements and their variability in individual organs confirmed by statistical analysis are also noteworthy. Findings from this study confirmed that the accumulation of a particular metal in a tissue or organ can reduce its concentration in another matrix or promote its increased content in another organ or tissue (Table 4). Red deer, as well as other wild animals, are closely linked to their natural habitat. They feed on vegetation growing in a given area of the habitat. The type of diet can strongly influence the concentration of individual elements in the tissues of these animals [5,6]. Therefore, game species, including red deer, can be used as bioindicators of environmental pollution [5,27]. On the other hand, meat, liver and kidneys are consumed by humans, and for this reason, the presence of harmful xenobiotics in these organs can pose a real threat to human health. Considering the above, the assessment of the safety and quality of food products sourced from game is very important [2,5,6,15]. 

The content of Fe, K, P, Cu, Zn and Ca in meat from deer is much higher than in other wild or livestock ruminants [1]. Findings from this study revealed that liver from red deer (Table 1) contained 200% more Fe in wet weight (when the percentage of water in the organ was considered), a comparable amount of Mn, 200% less Zn and 150% less Cu than the liver of fallow deer [16]. Considering macroelements, the liver of red deer contained 150% less Na, 50% less K and Ca and 300% less Mg (Table 2) compared to data reported for fallow deer by Vengušt and Vengušt [16]. Meat, liver and kidneys of red deer can be considered as an excellent source of essential elements such as Co, Cu, Mo, Mn, Se and Zn [28]. The comparison of levels of macro- and microelements in the meat, liver and kidneys of game species with the meat and other tissues of livestock shows that game meat is characterised by more beneficial proportions of these elements and better health-promoting properties [1,15,26]. Muscles from wild animals usually contain more Fe, Zn and Cu than those from livestock because wild animals are more physically active, and therefore, have higher levels of respiratory proteins containing Fe or Cu. The considerable variation in the content of macro- and microelements in individual tissues may result from many different factors. In addition to the aforementioned type of diet or physical activity, season of sampling, sex and age may also be of great importance [15,26]. Stags are hunted in the autumn–winter season, and therefore, a high concentration of most minerals in meat or other tissues may be related to the fact that after a few months of eating wholesome, natural food in spring and summer, these animals supplied greater amounts of essential nutrients to their tissues. The composition of macro- and microelements in muscles from red deer compared to muscles from roe deer, wild boar or hare [17] differed in some aspects between species. In this study, muscles from red deer contained 35% less Zn in wet weight compared to data reported by other researchers for muscles of roe deer or wild boar, and over 200% more Fe than muscles of roe deer, wild boar or hare. Cu is the main essential trace element, and its concentration in the liver, which is its main site of Cu accumulation and biotransformation, reflects the status of this element in the whole animal body. The content of Cu in the liver of red deer is usually lower than in the liver of fallow deer [1]. 

Considering the degree of accumulation of toxic metals, especially Pb and Cd, which pose the most serious hazard to health, this study revealed their different affinity for individual biological matrices (samples). The highest level of Pb was measured in the hair, followed by the *longissimus lumborum* muscle, liver, kidneys and testicles (Table 3). Slightly different concentrations of this metal in organs were reported by Jarzyńska and Falandysz [28], and it was highest in the liver, lower in muscles, and lowest in the kidneys of red deer. A slightly different trend in the accumulation of Pb was identified in tissues of fallow deer by Čelechovská et al. [29], and the level of Pb was lowest in the muscles, higher in the liver, and highest in the kidneys. In addition to environmental pollution with this element, a higher degree of accumulation, e.g., in muscles, could be attributed to Pb from lead bullets. According to Martin et al. [30], the meat of wild animals shot with this type of ammunition may contain increased levels of Pb. This creates an increased risk of humans consuming contaminated meat. The exposure may be directly related to the distance of organs from the gunshot wound; the effect of this factor may be minimised by careful collection of test matrices or organs intended for human consumption [31]. This study demonstrated that the concentrations of Pb in kidneys and liver calculated for wet weight were approximately 10–15% higher than concentrations reported for the organs of roe deer, red deer and wild boars from various regions of Poland [2]. In the cited study, levels of Pb ranged from 0.45 for roe deer liver and 0.50 mg·kg^−1^ w.w. for wild boar liver. In comparison, in the studies by Kasprzyk et al. (2020), the range for this metal in wild boar liver was from 0.16 to 0.26 mg kg^−1^ w.w. [15]. The levels of Pb in kidneys were slightly lower and ranged from 0.46 for roe deer to 0.53 mg·kg^−1^ w.w. for wild boar [2]. However, this study did not reveal any significant differences between the concentrations of this element in the analysed analogous biological matrices (Table 3). Slightly different findings were made with respect to the accumulation of Cd in tissues. The levels of Cd measured in this study tended to decrease in the following order: liver, kidneys, muscle, testicles and hair (Table 3). Čelechovská et al. [29] reported the highest concentration of Cd in kidneys, but lower concentrations in the liver and muscle of fallow deer, while in the study by Jarzyńska and Falandysz, it was highest in the kidneys and lower in the liver and muscle of red deer. In the case of red deer, these values were characterised by a significant scatter, from 0.22 to 12 mg·kg^−1^ of dry weight for muscles and kidneys, respectively [28]. In this study the scatter for Cd concentrations was much lower, and values ranged from 0.45 to 0.56 mg·kg^−1^ of dry weight for testicles and liver, respectively (Table 3). Higher values of Cd (in terms of dry weight), compared to those obtained in the own research in the liver of deer, were obtained by Kasprzyk et al. (2020) in the liver of wild boars, i.e., from 0.38 to 0.48 mg kg^−1^ w.w. [15]. The exception was the hair of red deer, in which Cd concentration was 50% lower (0.13 mg·kg^−1^ of d.w.) than in muscles of fallow deer. Animal hair is certainly one of the most important matrices that can be used as a bioindicator of trace elements in the environment [5,13], as with human hair [14]. The content of individual macro- and microelements as well as Pb and Cd in the hair of red deer was comparable to the content measured in the same matrix from red deer in the season before the present study [5]. This example confirms the effect of animal habitat on the status of these elements in the hair and other tissues. Particularly interesting is that the degree of environmental pollution in the central part of Poland did not differ significantly between the two study years [21]. The content of trace elements is usually analysed in the liver and kidneys, since these organs are the main accumulation sites of Zn, Cu and Cd due to metallothionein (MT) induction, a biomarker of exposure to Cd. Particularly remarkable is that the content of Cd in these organs is primarily associated with the composition of trace elements in water or food, while the concentration of Cd in muscles is mainly associated with animal activity [32]. Interestingly, despite the biomagnification of heavy metals in the food chain [4], their quantities are not always reflected in their higher concentration in animal tissues. For example, the fox is at the top of the food chain in Poland, and thus, is potentially more exposed to high Cd levels, but very low concentrations of this element were found in its liver (0.13 ± 0.03 mg·kg^−1^ of w.w.) and kidneys (0.26 ± 0.06 mg·kg^−1^ of w.w.) [32]. Cadmium levels measured by other researchers were similar (in the liver) and slightly higher (in the kidneys) compared to levels found in this study for these organs in red deer. Reportedly, testicles are a specific matrix for the measurement of concentration of individual elements. Heavy metals, especially Cd, can induce, for example, dystrophic calcification of testicles, which is usually associated with disturbed mineral homeostasis in the whole body, increased antioxidant enzyme activity and protein oxidation. Deficiency or excess of minerals can lead to testicular damage at the microstructural level, as well as cause various dysfunctions [33]. 

This study revealed a significant negative correlation between Cd and Ca in testicles (r_xy_ = −0.743; *p* ≤ 0.01), which supports the claim by Cupertino et al. [33] that Cd can compete with Ca and cause various disorders of the reproductive system. Moreover, Cd interferes with Ca metabolism, which may have a direct toxic effect on bones and an indirect impact on the kidneys, with possible nephrotoxicity [34]. In this research, a negative relationship was demonstrated between the metals in the kidneys. Moreover, Cd influences iron metabolism. The higher the blood Cd level, the lower the ferritin level [10,34]. This research showed such an inverse relationship between these elements in the liver (Table 7). Interactions between Pb and other toxic and essential metals are also very important and depend on many factors, including a degree of exposure to heavy metals which penetrate into living organisms from the external environment, as well as the concentrations of these metals in tissues probably associated with their different functions, type of fibre, and thus, a different degree of biotransformation or metabolism of these elements. Animals exposed to Pb usually have a lower Cu content in the liver [34], which was confirmed by this study (Table 7). An inverse correlation was also found between these elements in the testicles (Table 6). These interactions may depend on the relative concentrations of the compounds and the storage tendency/affinity of these metals in particular tissues. Particularly remarkable is also a significant negative correlation between Zn and Cu in the hair (Table 5). The same negative correlation (r_xy_ = −0.71; *p* ≤ 0.01) was reported from similar studies on the hair of red deer by Cygan-Szczegielniak et al. [5] and for the liver of wild boar (r_xy_ = −0.201; *p* ≤ 0.05) by Neil et al. [18], which may prove that these elements compete with each other to be absorbed from the gastrointestinal tract. Multiple correlations between metals and between metals and other structural compounds can influence the physiology of living organisms and even cause various dysfunctions. Long-term exposure to relatively low concentrations of metals, especially Cd, may lead to interactions with lipid peroxidation products, especially in muscles, liver or kidneys, and even have a toxic effect on many systems because of Cd binding to biological structures [35]. This research confirmed several statistically significant correlations between the same element in different matrices (Table 4). In this case, the largest share in these relationships is ascribed to the hair and liver, as well as to the hair and kidneys. This may indicate that the hair is a good matrix for the initial assessment of animal exposure to various metals, which was confirmed, among others, by Cygan-Szczegielniak et al. [5,13] and other authors [3,14]. Moreover, the hair may be collected from the animal in vivo, which in some cases is of great importance. 

## 5. Conclusions

The study revealed high variability and many significant correlations between the levels of mineral elements and selected toxic metals in tissues, organs and hair of red deer. Statistically significant correlations, between macro and microelements, were mostly noted in the hair. Additionally, statistically significant correlations between toxic metals and macro and microelements were confirmed mainly in the liver, kidneys and testicles. Statistically significant relationships were also found between the same element in different matrices. Additionally, in this case, hair vs. liver and hair vs. kidneys had the largest share in the relationships. Pb and Ca were characterised by the greatest variability among all the elements, regardless of the measurement matrix. Findings from this study confirmed that the accumulation of a specific metal in a tissue or organ can reduce its concentration in another matrix or promote its increased content in another tissue or organ. However, this part of the research should be supplemented with further analyses in this area in order to clarify these relationships. 

Understanding the relationships between elements in the tissues may be useful for the assessment of various abnormalities that occur in living organisms. The monitoring of toxic metals in specific organs can provide valuable information about the pollution of the animal habitats, and about their exposure to these pollutants.

## Figures and Tables

**Table 1:** The variability factor and content of Mn, Zn, Cu (mg·kg^−1^ of d.w.) and Fe (g·kg^−1^ of d.w.) in tissues and organs of red deer.

Mineral Elements	Research Matrices																			
	Testicles				Liver				Kidneys				Muscle				Hair			
	$\bar{x}$	Me	SE	CV	$\bar{x}$	Me	SE	CV	$\bar{x}$	Me	SE	CV	$\bar{x}$	Me	SE	CV	$\bar{x}$	Me	SE	CV
Fe g·kg^−^^1^	0.393 ^A^	0.383	±0.022	17.40	0.762 ^B^	0.751	±0.008	3.24	0.351 ^A^	0.356	±0.008	7.63	0.297 ^A^	0.292	±0.014	14.64	0.259 ^A^	0.247	±0.019	22.97
Mn mg·kg^−^^1^	6.57 ^A^	6.51	±0.213	10.24	9.69 ^B^	9.46	±0.184	5.99	10.42 ^B^	10.37	±0.173	5.27	7.71 ^A^	7.65	±0.132	5.41	19.32 ^C^	19.30	±0.162	2.66
Zn mg·kg^−^^1^	130.8 ^A^	127.1	±0.591	11.10	43.18 ^B^	42.41	±0.810	5.93	125.7 ^A^	125.8	±1.693	4.26	71.11 ^C^	71.05	±2.911	12.94	144.3 ^A^	145.4	±1.709	3.75
Cu mg·kg^−1^	17.79 ^A^	17.93	±0.471	8.37	38.42 ^B^	38.52	±0.194	1.60	16.70 ^A^	16.71	±0.190	3.60	43.41 ^B^	43.45	±0.418	3.05	17.66 ^A^	17.88	±0.192	3.44

^A,B,C^—values marked with different letters in the same row differ significantly (*p* ≤ 0.01), which was analysed using the Kruskal–Wallis test; Me—Median (Q_2_); SE—standard error; CV (%)—coefficient of variation.

**Table 2:** The variability factor and content of selected macroelements in tissues and organs of red deer (g·kg^−1^ of d.w.).

Mineral Elements	Research Matrices																			
	Testicles				Liver				Kidneys				Muscle				Hair			
	$\bar{x}$	Me	SE	CV	$\bar{x}$	Me	SE	CV	$\bar{x}$	Me	SE	CV	$\bar{x}$	Me	SE	CV	$\bar{x}$	Me	SE	CV
Na g·kg^−1^	3.61 ^ac^	3.57	±0.149	13.04	0.939 ^b^	0.958	±0.015	5.21	2.50 ^b^	2.46	±0.052	6.62	2.58 ^b^	2.57	±0.126	15.51	3.43 ^c^	3.46	±0.089	8.27
K g·kg^−1^	6.45 ^A^	6.67	±0.245	12.03	1.54 ^B^	1.55	±0.021	4.37	5.51 ^A^	5.48	±0.078	4.52	14.06 ^C^	13.96	±0.279	6.29	1.15 ^B^	1.11	±0.048	13.12
Ca g·kg^−1^	0.145 ^a^	0.146	±0.007	14.36	0.121 ^a^	0.102	±0.016	43.19	0.115 ^a^	0.094	±0.026	63.40	0.187 ^a^	0.151	±0.035	59.72	1.80 ^b^	1.99	±0.181	31.78
Mg g·kg^−1^	0.454 ^a^	0.441	±0.04	27.94	0.154 ^b^	0.151	±0.004	8.92	0.564 ^a^	0.561	±0.021	12.09	1.43 ^c^	1.45	±0.029	6.41	0.661 ^a^	0.665	±0.033	15.84

^a, b, c, d^—values marked with different letters in the same row differ significantly (*p* ≤ 0.05); ^A,B,C,D^—values marked with different letters in the same row differ significantly (*p* ≤ 0.01), which was analysed using the Kruskal–Wallis test; Me—Median (Q_2_); SE—standard error; CV (%)—coefficient of variation.

**Table 3:** The variability factor and content of Pb and Cd in tissues and organs of red deer (mg·kg^−1^ of d.w.).

Toxic Metals	Research Matrices																			
	Testicles				Liver				Kidneys				Muscle				Hair			
	$\bar{x}$	Me	SE	CV	$\bar{x}$	Me	SE	CV	$\bar{x}$	Me	SE	CV	$\bar{x}$	Me	SE	CV	$\bar{x}$	Me	SE	CV
Pb mg·kg^−1^	2.84 ^A^	2.61	±0.203	22.63	2.59 ^A^	2.46	±0.109	13.29	2.65 ^A^	2.61	±0.085	10.13	5.37 ^B^	5.11	±0.815	48.00	7.57 ^C^	7.28	±0.32	13.37
Cd mg·kg^−1^	0.454 ^A^	0.471	±0.011	7.57	0.565 ^B^	0.561	±0.015	8.23	0.544 ^B^	0.531	±0.021	12.36	0.491 ^A^	0.517	±0.041	26.19	0.129 ^C^	0.123	±0.004	9.46

^A,B,C,D^—values marked with different letters in the same row differ significantly (*p* ≤ 0.01), which was analysed using the Kruskal–Wallis test; Me—Median (Q_2_); SE—standard error; CV (%)—coefficient of variation.

**Table 4:** Strong significant correlations between the concentrations of the same elements in different organs and tissue—interaction coefficients.

Mineral Elements/ Research Matrices	Na (Liver)	Fe (Hair)	Ca (Muscle)	Mg (Hair)	Mn (Hair)	Cu (Hair)	Pb (Hair)	Cd (Hair)
Na (kidneys)	−0.708 **							
Fe (kidneys)		0.889 **						
Ca (kidneys)			−0.757 **					
Mg (kidneys)				−0.734 **				
Mn (kidneys)					0.656 **			
Cu (kidneys)						0.845 **		
Pb (liver)							−0.745 **	
Cd (liver)								−0.768 **

Interaction coefficients are significant at *p* ≤ 0.01 **.

**Table 5:** Correlation coefficients (r_xy_) for the levels of selected mineral elements and toxic metals in the hair of red deer.

Mineral Elements	K	Fe	Ca	Mg	Mn	Zn	Cu	Pb	Cd
Na	0.760 **	−0.134	0.061	0.253	−0.535	0.547	−0.311	0.158	−0.274
K		0.273	−0.091	0.122	−0.285	0.224	0.073	0.285	0.097
Fe			0.079	−0.553	0.576	−0.115	0.687 *	0.697 *	0.292
Ca				−0.091	0.091	0.709 **	−0.201	0.127	−0.085
Mg					−0.571	0.322	−0.439	−0.067	0.207
Mn						−0.2	0.693 *	0.20	0.201
Zn							−0.682 *	0.152	−0.103
Cu								0.213	0.274
Pb									−0.043

Correlation coefficients were significant at *p* ≤ 0.05 *, *p* ≤ 0.01 **.

**Table 6:** Correlation coefficients (r_xy_) for the levels of selected mineral elements and toxic metals in the testicles of red deer.

Mineral Elements	K	Fe	Ca	Mg	Mn	Zn	Cu	Pb	Cd
Na	0.222	0.440	0.258	−0.211	0.061	0.470	0.421	−0.251	−0.396
K		0.554	0.160	0.400	0.191	−0.104	0.252	−0.357	0.249
Fe			−0.156	0.180	0.223	0.535	0.650 *	−0.488	0.420
Ca				−0.183	−0.318	0.232	−0.329	0.281	−0.743 **
Mg					−0.027	−0.061	0.413	−0.463	0.123
Mn						−0.377	0.043	−0.247	0.235
Zn							0.309	0.097	−0.098
Cu								−0.863 **	0.271
Pb									−0.457

Correlation coefficients were significant at *p* ≤ 0.05 *, *p* ≤ 0.01 **.

**Table 7:** Correlation coefficients (r_xy_) for the levels of selected mineral elements and toxic metals in the liver of red deer.

Mineral Elements	K	Fe	Ca	Mg	Mn	Zn	Cu	Pb	Cd
Na	−0.465	0.347	0.183	0.001	0.263	−0.263	0.251	0.177	0.405
K		−0.238	−0.654 *	−0.036	0.006	0.248	0.455	−0.424	−0.207
Fe			0.069	0.063	0.138	−0.532	−0.344	0.594	−0.651 *
Ca				0.085	−0.091	0.006	−0.612	0.418	−0.085
Mg					0.225	0.073	−0.462	−0.043	0.512
Mn						0.091	−0.139	0.030	0.140
Zn							0.164	0.042	0.195
Cu								−0.627 *	0.155
Pb									−0.176

Correlation coefficients were significant at *p* ≤ 0.05 *.

**Table 8:** Correlation coefficients (r_xy_) for the levels of selected mineral elements and toxic metals in the kidneys of red deer.

Mineral Elements	K	Fe	Ca	Mg	Mn	Zn	Cu	Pb	Cd
Na	−0.780 **	−0.043	−0.579	−0.227	0.196	0.024	−0.043	0.488	0.267
K		−0.308	0.353	0.095	−0.232	0.152	−0.340	−0.243	−0.315
Fe			−0.117	0.549	0.135	0.153	0.595	0.166	0.562
Ca				0.061	0.395	−0.212	0.491	−0.127	−0.695 *
Mg					0.517	0.476	0.409	0.354	0.503
Mn						0.255	0.693 *	0.498	−0.098
Zn							0.200	0.176	0.189
Cu								0.184	−0.085
Pb									0.421

Correlation coefficients were significant at *p* ≤ 0.05 *, *p* ≤ 0.01 **.

**Table 9:** Correlation coefficients (r_xy_) for the levels of selected mineral elements and toxic metals in the muscle of red deer.

Mineral Elements	K	Fe	Ca	Mg	Mn	Zn	Cu	Pb	Cd
Na	0.073	−0.370	−0.479	−0.006	−0.055	0.139	0.067	−0.479	−0.406
K		0.176	−0.164	−0.037	−0.395	−0.310	0.274	−0.213	−0.517
Fe			0.139	−0.584	−0.382	−0.358	0.588	0.018	−0.321
Ca				0.091	−0.479	−0.164	−0.176	0.200	0.224
Mg					0.024	0.450	−0.049	0.389	0.091
Mn						−0.006	−0.345	−0.261	0.648 *
Zn							0.067	0.418	0.139
Cu								0.030	−0.564
Pb									−0.103

Correlation coefficients were significant at *p* ≤ 0.05 *.

## Data Availability

The data presented in this study are available on request from the corresponding author.
